# Are Molecularly Imprinted Polymers (MIPs) Beneficial in Detection and Determination of Mycotoxins in Cereal Samples?

**DOI:** 10.22037/ijpr.2020.112677.13887

**Published:** 2020

**Authors:** Dara Hatamabadi, Bahar Mostafiz, Amirreza Dowlati Beirami, Kamran Banan, Niloufar Sharafi Tafreshi Moghaddam, Hanif Afsharara, Rüstem Keçili, Fatemeh Ghorbani-Bidkorbeh

**Affiliations:** a *Department of Medicinal Chemistry, School of pharmacy, Shahid Beheshti University of Medical Sciences, Tehran, Iran. *; b *Department of chemistry, Faculty of Physics and Chemistry, University of Alzahra, Vanak, Tehran, Iran. *; c *Department of Pharmaceutics, School of Pharmacy, Shahid Beheshti University of Medical Sciences, Tehran, Iran. *; d *Yunus Emre Vocational School of Health Services, Department of Medical Services and Techniques, Anadolu University, Eskişehir, Turkey.*

**Keywords:** Cereal, Molecularly Imprinted Polymer, Detection, Determination, Mycotoxins

## Abstract

The process of matrix clean-up and extraction of analytes has a significant influence on the detection and determination of the analyte, especially in trace amounts. Molecularly imprinted polymers (MIPs) are solid particles that can absorb specific molecules regarding the template molecule used in the synthesis process of each type of MIP. As a result, they can be used in more effective and more specific solid-phase extraction processes. On the other hand, mycotoxins are second metabolites of molds and fungus which are potentially cytotoxic and/or genotoxic even in trace amounts, and due to extensive consumption of cereals and the great concern of public health, several methods were developed and currently are in the process of development to detect and determine the presence and amounts of mycotoxins in cereals. This review is aimed to investigate the application and efficacy of MIPs in detecting and determination of mycotoxins in cereals.

## Introduction

The deleterious effects of several food contaminations on human and animal health have resulted in the ongoing development of analytical methods for enhanced detection and determination of food contaminations (1). Cereals are a group of nutrients consumed by billions of people which may contain toxic fungal metabolites such as mycotoxins as their natural contaminations (2, 3). Conventional methods such as high-performance liquid chromatography (HPLC), liquid chromatography/mass spectrometry (LC-MS), liquid chromatography-tandem mass spectrometry (LC-MS/MS), gas chromatography-mass spectrometry (GC-MS), and enzyme-linked immunosorbent assay (ELISA) have been used for the detection and determination of mycotoxins in cereals (4-7). Extraction and clean-up methods (*e.g*., Solid-phase extraction methods) are important in the determination of trace amounts of mycotoxins (4, 8). This article reviews the application of molecularly imprinted polymers (MIPs) in the detection and/or determination of mycotoxins in cereals.

One of the approaches in utilizing imprinted polymers is through sensor technology. Among different types of sensors, electrochemical sensors are one of the most investigated sensors in the analysis of drugs and toxins (9-11). Therefore, the combinatorial application of imprinted polymer and these sensors is also amongst hot topics in the analytical chemistry field. There have been an extensive number of researches focused on using electrochemical sensors for the detection and analysis of mycotoxins. However, due to the presence of numerous recent reviews around this subject (12, 13), we have excluded electrochemical sensors from scopes of this review and only have focused on conventional analysis methods.

*Cereals and mycotoxins*

Cereals are defined as plants from grass family which produce edible seeds or grains (14). Cereals and grains contain complex carbohydrates, so they are a considerable part of the food pyramid in daily regimens (15).

The Food and Agriculture Organization (FAO) has categorized primary cereals to seventeen groups based on their genus, species and other factors. These seventeen groups are Wheat, Rice, Barley, Maize, white maize, Popcorn, Rye, Oats, Millets, Sorghum, Buckwheat, Quinoa, Fonio, Triticale, Canary Seed, Mixed Grain (grains which are harvested from plants with two or more genera), and cereals (16).

Twenty-five percent of crops all around the world are estimated to have been affected by molds, and mycotoxins are secondary metabolites of them (mostly Fusarium Spp. Aspergillus Spp., Penicillium Spp.) (17). Until 2017 more than 500 mycotoxins have been identified and reported (18, 19).

During the years, our knowledge about mycotoxins has been developed. The major mycotoxins which are considered in legislation of agriculture products (or regulating systems) are: aflatoxin (Produced by Aspergillus spp.) (20-22), ergot alkaloids (mainly produced by Claviceps Spp.) (23), citrine (produced by *Penicillium* spp., Monascus spp., and *Aspergillus* spp.) (24-26), patulin (produced by *Penicillium* spp. and *Aspergillus* spp.) (27), fumonisins and zearalenone (produced by *Fusarium* spp.) (28, 29), trichothecenes (produced by various species of *Fusarium*, *Myrothecium*, *Trichoderma*, *Trichothecium*, *Cephalosporium*, *Verticimonosporium*, and *Stachybotrys*.) (30-32), ochratoxin A (produced by *Aspergillus* spp. and *Penicillium* spp.) (33, 34). The toxicity of mycotoxins may range from human genome damage which may result in cancer (35) to acute or chronic toxicity in humans and chronic toxicity is more common (17).

Aflatoxin B1 is known for having the strongest carcinogenicity effect among all mycotoxins (36, 37). Molds can be contaminating agents for cultivation products before or after harvesting and in the storing stage (38).

Accumulations of mycotoxins in cereals can become a great danger for human health. In the long term view, exposure to mycotoxins can cause considerable economic losses (39). Standard limits for each mycotoxin need to be set in order to control the health issues caused by mycotoxins. The standard limits for mycotoxins in foods and feedings are regulated by the Codex Committee on Food Additives and Contaminants (CCFAC). CCFAC has established limits and standards for mycotoxins in processed and unprocessed foods globally (40).

*Molecularly imprinted polymers*

Clearly, every molecule has a unique stereochemistry (41). There is only one way to find the Known fairy tale Cinderella by matching her glass shoes. If the unknown molecule is the missing princess, MIPs would be the unique glass shoe. Introducing the best way of sensing a molecule is fundamental for tracing an analytical process. In other words, molecularly imprinted polymers are synthetic antigen-antibody analogs. Through the interactions between the template and imprinted polymer, strong and specific bonds emerge. The strength of the interactions between different sets of templates and MIPs may differ based on the number and strength of bonds.

Due to the formation of a unique set of bonds between the template and imprinted polymer, MIPs are potentially selective and specific platforms for detecting the template and template-like molecules in various sample matrices (42-44).

History of MIPs began at 1930 when Polyakov (45) and Dickey (46) reported that silica matrix amasses together at the presence of small molecules and show affinity toward these molecules. In 1973 Wulff proposed a new concept “Enzyme-Analogue Built Polymers” and refreshed the idea of molecular imprinting (47, 48). However, the term “Molecular Imprinted Polymer” was stated by Mosbach and Sellergren in 1984 (49, 50). These were very first steps of MIPs, and they opened a new door to different aspects of polymer science. Mosbach, *et al*. mainly focused on recognition of non-covalent bonds between the template and imprinted polymer, (51, 52), while Wulff, *et al*., were researching in the opposite direction and mainly tended to create covalent bonds between the template and Imprinting Polymer (53). The difference between these two methods is in their chemical base; through the covalent synthesis method, homogeneous rebinding cavities emerge and therefore make more selective and effective MIPs. Wulff used MIPs in catalytic reactions, and Mosbach used MIPs to develop a separation method for sensing various analyte (44, 55).

*Fundamentals of MIPs*

Imprinting technique is mainly based on the fact that a template molecule (the target molecule) or dummy-template molecule (a similar molecule to the target molecule) is also present in the polymerization matrices, and during the reaction, the template is surrounded by the synthesized polymer, and after the endpoint of the reaction, the template gets washed out of the polymer and results in a porous polymer with cavities similar to the stereochemistry of the template (56).

The formation of MIPs is divided into three different manners. The most prevalent method is the non-covalent method, mostly based on hydrogenic, ionic, Van der Waals, and π-π interactions between the template and forming polymer or monomers, and makes it rapid, straightforward, and the most common. (57). However, in the covalent method the template molecule at first conjugates with a specific monomer and then renewed monomer goes through the polymerization process. This method is less favorable than the previous method due to the requirements of reversible condensation reactions which are limited and cannot be applied to a wide range of analytes. This method has also a low rate of bond formation, and the dissociation of the polymer and template is dependent on thermodynamic conditions (58).

**Figure 1:**
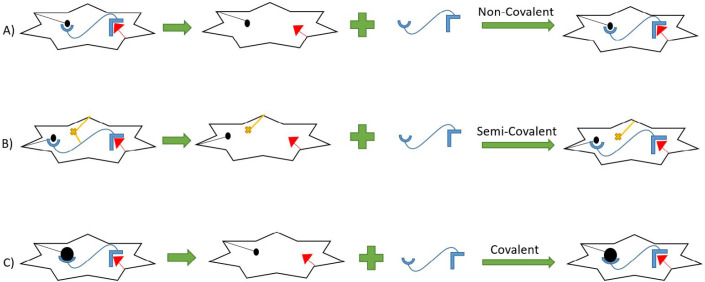
Different procedures for MIP synthesis (A) Non-Covalent synthesis (B) Semi-Covalent Synthesis (C) Covalent synthesis

The third method is a combination of the latter two ones. The process of polymerization is mainly based on the formation of covalent bonds. However, through non-covalent bonds, rebinding, and removal of the template will take place (59). (Figure 1). A template is a molecule with a unique stereochemistry and cavities inside the polymer, which mimic the conformation and configuration of the original molecule. . Generally, template and analyte are the same, but there may be some challenges*, *For example the original analyte is unstable and decomposes through the polymerization process or there is an incompatibility between the template and the other components present in the matrices. Therefore, a similar molecule is represented as the template, which mimics some of the template specific properties. Consequently, the created cavities have similarity to the analyte. These new templates are called dummy molecules or dummy templates.

Characteristics such as having stability through the polymerization process, and proposing specific functional groups which can interact or react with the monomers are required for template molecule. The template needs to have interference with the polymerization process (60).

The template molecules can be classified into three major categories. The first and the predominant type of templates are small organic molecules which are usually stable and contain specific functional groups. Most of the organic pollutants, pharmaceuticals, and pesticides whose extraction and detection are of high value, are categorized in this category. The second one is non-organic molecules, usually metals, which get imprinted in their ionic forms and they are called “ionic imprinting polymers” (IIP) instead of “Molecularly imprinted polymers” (MIP). The overall process is different and harder in these cases as monomers are required to be chelating agents, and the selectivity of these polymers is usually low as metal ions often possess similar physicochemical properties. The third type of templates is biological macromolecules. Due to their large and complex structures and susceptibility of their conformations, conventional methods cannot be practiced on them. However, recently some new methods have been introduced (61-66).

The very first step of MIP synthesis is determining a proper monomer that shows the best interaction with the template molecule in the pre-polymerization step. Mainly through the non-covalent method, hydrogen (donor or acceptor bonds) bonds emerge. On the other hand, Methacrylic Acid (MAA) contains a carboxylic acid functional group, so it is both donor and acceptor of hydrogen bonds. This made MAA the most common monomer in free radical polymerization (56, 67).

The other essential agent is crosslinker monomer which works as the rigidifying agent, and fixes the monomers around the template. Therefore, the shape of cavities and the position of functional groups in the polymer remain the same even after the removal of the template. The molecule which is used as the crosslinker is vital as it dictates the flexibility and the rigidity of the polymer. Even the concentration of the crosslinker is also important, since high amounts result in a decrease in the amount of the recognition sites, and low amounts result in mechanical instabilities in the polymer (67).

**Figure 2:**
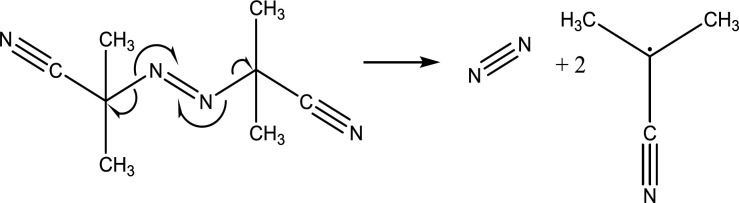
Mechanism of Radical Polymerization initiation

Initiation is a mandatory step for the beginning of the chemical process of polymerization. The most commonly used reaction is free-radical based ones in which the polymerization starts with the cleavage of the initiator’s azo or peroxide bonds due to sufficient thermal or ultra violet (UV) photonic activation energies. Free radical forms created through this process attack the monomer or the crosslinker vinyl groups due to its electrophilic properties. Azoisobutylnitrile (AIBN) is the predominant free radical polymerization initiator (Figure 2) (68).

Porogen is the solvent which works as the dispersion medium for the components of the polymerization process. The most critical parameter of the porogen is the polarity of the solvent. As the polarity of the solvent increases its interference with functional groups of the polymerization components and the template also increases, and it results in cavities that are less similar to the template molecule and, therefore, the sensitivity and adsorption capacity of the polymer decreases. Among common porogens, Dimethylsulfoxide (DMSO), Acetonitrile, Chloroform, N,N-dimethylformamide (DMF), and toluene are mentioned (69, 70).

*Approaches*

There are different methods for the synthesis of molecularly imprinted polymers. Among these methods, bulk polymerization, suspension polymerization, precipitation polymerization, and surface-modified polymerization are commonly used.

Bulk polymerization is the most conventional and practiced method of MIP polymerization. Through this method, polymerization occurs in a solution, and a block of polymer is acquired; furthermore, the block is ground and sieved, which results in non-uniform particle size and shape. Throughout the grinding, some of the active sites may get destroyed, and throughout the sieving, some of the polymers are lost (71, 72).

On the other hand, suspension polymerization is almost as simple as bulk polymerization. In this method, the solution is more diluted than bulk polymerization, and it results in uniform spherical polymers (73).

Precipitation polymerization is based on the coagulation of nanogels into uniform spherical particles, and these particles grow into larger ones by capturing oligomers in the solution. This method has a higher yield, but the downside is that it also requires higher amounts of template due to the low concentration of the solution (74).

Another method is surface modified polymerization. In this method, firstly, a core is synthesized which is usually getting coated by a thin layer of silica, and afterward, a thin layer of polymer is also getting coated on it. Due to the low thickness of the polymer, wash and adsorption processes are more efficient, and it usually results in high efficiency and low amount of template bleeding (75). In this method the surface or the core can be modified in variety of ways which make these particles to be useful in different systems and applications such as sensors, Drug delivery, and separation (76, 77).

*Determination of mycotoxins using MIP*

In 2006, Urraca, *et al*. used MIPs for clean-up and detection of Zearalenone and its metabolite, α-Zearalenol, in several types of cereals and swine feed samples. They used a rationally designed zearalenone analog, cyclododecyl 2,4 di hydroxybenzoate (CDHB), as a dummy template and HPLC with a fluorescent detector for analysis. Limit of detections (LODs) via this procedure was lower than accepted MRL, and similar to LODs reported using immunoaffinity columns and LC-MS (78).

De Smet synthesized a Fumonisin B1 MIP for extraction of Fumonisin B analogs in rice, bell paper, and cornflake samples. The cross-reactivity study results demonstrated that these MIPs are specific to Fumonisin B (FB) analogs and do not offer retention capabilities for other types of mycotoxins except for Ochratoxin A (79).

In 2010, a study compared the crushed monolith and micro-bead Ochratoxin A MIPs in order to extract Ochratoxin A from wheat samples. This evaluation indicated that the micro-bead format MIPs had stronger retention capabilities. They also compared Ochratoxin A immunoaffinity cartridges with MIP beads. The results showed that unlike immunoaffinity chromatography (IAC), MIP beads are tolerant of overloading and saturation due to significantly higher capacity (80).

De Smet, *et al.* also synthesized a MIP for selective detection of T-2 toxin in 3 types of cereals: maize, barley, and oat. They used T-2 toxin as the template through bulk polymerization procedure and compared the results with IAC and OASISHLB® column clean-up methods. This comparison demonstrated that recovery rates obtained using OASISHLB® columns were higher than those obtained with IAC and MIP. However, in the case of the LOD and limit of quantification (LOQ), MIPs offered the lowest LOD and LOQ, which is more suitable for the detection and quantification of T-2 toxin in grain samples (81).

Lucci, *et al*. made an imprinting polymer cartridge for the extraction of Zearalenone based on the concept previously used for other molecules, *i.e*., AFFINIMIPTM. A dummy molecule, cyclododecyl 2,4-dihydroxybenzoate (CDHB) was chosen as the template based on the previous works of Urraca,*. et al*. and the capability of these polymers in the absorption of Zearalenone in wheat and corn samples were measured, and the recoveries ranging from 82%-90% were also demonstrated (78, 82). The capacity of these imprinted polymers was also compared with ZearalatTest™, which is an immunoaffinity based column. Due to the saturation of active sites in ZearalatTest™, Zearalenone could not be measured accurately in the amount of higher than 2000 µg/kg. On the other hand, ZearalatTest™ resulted in much cleaner chromatograms. They also determined the selectivity of these polymers using a cross-reactivity test by α-Zearalenol, a toxic metabolite of Zearalenol which is really similar to Zearalenol and Ochratoxin A. It was shown that these polymers could also absorb α-Zearalenol and it showed a high recovery between 87%-93%. However, the Ochratoxin A was not absorbed by the polymer at all which shows this polymer has high specificity toward Zearalenol and α- Zearalenol (83).

Lenain, *et al.* (2012) produced a general imprinted polymer for the extraction of ergot alkaloid from barley. They used Metergoline as their template and assessed the ability of their polymer in the extraction of 12 ergot alkaloids, ergotamine (Et), ergotaminine (Etn), ergocornine (Eco), ergocorninine (Econ), ergocryptine (Ekr), ergocryptinine, (Ekrn), ergocristine (Ecr), ergocristinine (Ecrn), ergosine (Es), ergosinine (Esn), ergometrine (Em), and ergometrinine (Emn). Recoveries ranged from 56%-79%. Although the recoveries are not as high as other imprinted polymers, due to using a dummy molecule to represent 12 alkaloids, it is almost in the acceptance range of European commission (EC) 401/2006 guideline which is 60% to 120%. This research group used two different synthesis methods including suspension polymerization, which resulted in almost uniform and spherical polymers and the bulk synthesis which resulted in amorphous polymers, and they showed that polymers obtained from suspension polymerization method are far more efficient than the bulk. Different varieties of washing solvents were also studied and it was found out that the best solvent, which resulted in the most diminishment of the matrix effect, is water. Furthermore, in the cross-reactivity test, 25 common mycotoxins were tested, and the compounds were categorized into three groups of low, medium, and high cross-reactivity (84).

Díaz-Bao, *et al*., in 2016, attempted creating an imprinted polymer for the extraction of 5 Aflatoxins (M1, B1, B2, G1, G2) from Cereal based Babies’ foods. They used 5,7-Dimethoxycoumarin as a dummy template molecule, and they made these polymers around magnetic beads so an external magnet could easily collect them. However, the recovery results were not satisfactory, and they only got 39%-44% recovery. The team concluded that these results were due to the use of a dummy molecule and the matrix effect. The team suggested that these results show the possibility of making imprinted polymers for aflatoxins, but theirs is not to substitute conventional methods in practice (85).

Hu, *et al*. produced a core-shell structure with a magnetic core (ferroferric oxide) and a polydopamine-based molecularly imprinted polymer shell as a method for extraction of different types of ochratoxins (ochratoxin A, ochratoxin B, and ochratoxin C). The template molecule used to form the polymer cavities for all three types of ochratoxins was ochratoxin A. Polymerization process was carried out in Tris-HCl buffer pH 3.0. No crosslinkers were mentioned in the polymerization process. The recovery percentage was reported in rice samples as 71.0%. Cross-reactivity of Aflatoxin B1, fumonisin B1, and Zearalenone was tested to check the affinity of MIP synthesized in this study. In a similar concentration of ochratoxin, peak area of Aflatoxin B1 and fumonisin B1 was less than ± 5%, and peak area of Zearalenone was less than ± 2% of the original peak area (86).

Huang, *et al*. developed a MIL-101@molecularly imprinted polymer core-shell for extraction of Zearalenone in three grains (corn, wheat, and rice). The functional monomer was MMA, coumarin-3-carboxylic acid acted as a dummy molecule, and ethylene glycol dimethacrylate hydroxyethyl methacrylate was used as a crosslinker in the polymerization process. After the production of MIL-101@MIPs, they were packed into a self-made cartridge. Solid phase extraction (SPE) optimization was done to increase the response, and three different linear ranges were obtained for each grain, all between 6.25 to 250 μg per kg. Recoveries ranged from 82.0 to 84.8% (87).

Recently, Lhotská, *et al*. prepared selective citrinin molecularly imprinted polymer. The structural monomer was acrylamide, and as for dummy molecule, 1-hydoxy-2-naphtoic acid was chosen due to the toxicity of citrinin. After the preparation of MIP and non-imprinted polymer (NIP), both were packed into steel cartridges and coupled to the on-line SPE-HPLC system. The SPE conditions were optimized and the application in the real sample was investigated in barley and wheat specimens. Recovery achieved in this study reached to 82.0% with a relative standard deviation (RSD) of 1.5% (88). 

Munawar *et al*. have introduced a new method for detection and extraction of Fumonisin B1 (FB1) mycotoxin in maize. Their paper suggests a developing molecularly imprinted polymer nanoparticle based assay (MINA) method. In this work, FB1-derivatized glass beads were produced, and after preparing a mixture of 5 monomers in polymerization solution, the polymerization was done on the surface of mentioned glass beads. Therefore, the derivatized beads acted as a template molecule. Then they are all packed into a cartridge. Molecularly imprinted polymer nanoparticle based assay (MINA) was compared with HPLC and ELISA method and showed a dilution factor of 80% compared to 20 and 5% of HPLC and ELISA, with recovery of 108-113% (89).

Rui, *et al*. prepared a selective MIP in the form of a core-shell for extraction and enrichment of 4 different types of aflatoxins (G1, G2, B1, and B2). FDU-12@MIPs were synthesized first by preparing and modifying of FDU-12 and then dispersing it in a solution of 7-acetoxy-4-methylcoumarin as the monomer, MAA and EDMA as crosslinkers and azo-butyronitrile as initiator. FDU-12@NIPs were also prepared under the same condition but without the monomer. Three grains responded to this method (wheat, rice, and corn) with recovery rang of 82.6–116.7% and RSD of 2.73 -4.21% (90).

All synthesis and analytical method validation information are summarized in Tables 1 and 2.

**Table1:** Synthesis data of MIPs used in determination of mycotoxins in cereal

**Analyte**	**Matrix**	**Template**	**MIP synthesis**	**Monomer**	**Crosslinker**	**Porogen**	**M/CL/T ratio**	**extraction-analytical method**	**Ref**
α-zearalenol	Swine feed	CDHB	Bulk	1-ALPP	TRIM	Acetonitrile	4:20:01	PLE - HPLC(fluorescence detector)	(78)
α-zearalenol	Wheat	CDHB	Bulk	1-ALPP	TRIM	Acetonitrile	4:20:01	PLE - HPLC(fluorescence detector)	(78)
α-zearalenol	Corn	CDHB	Bulk	1-ALPP	TRIM	Acetonitrile	4:20:01	PLE - HPLC(fluorescence detector)	(78)
α-zearalenol	Barley	CDHB	Bulk	1-ALPP	TRIM	Acetonitrile	4:20:01	PLE - HPLC(fluorescence detector)	(78)
α-zearalenol	Rye	CDHB	Bulk	1-ALPP	TRIM	Acetonitrile	4:20:01	PLE - HPLC(fluorescence detector)	(78)
α-zearalenol	Rice	CDHB	Bulk	1-ALPP	TRIM	Acetonitrile	4:20:01	PLE - HPLC(fluorescence detector)	(78)
zearalenone	Swine feed	CDHB	Bulk	1-ALPP	TRIM	Acetonitrile	4:20:01	PLE - HPLC(fluorescence detector)	(78)
zearalenone	Wheat	CDHB	Bulk	1-ALPP	TRIM	Acetonitrile	4:20:01	PLE - HPLC(fluorescence detector)	(78)
zearalenone	Corn	CDHB	Bulk	1-ALPP	TRIM	Acetonitrile	4:20:01	PLE - HPLC(fluorescence detector)	(78)
zearalenone	Barley	CDHB	Bulk	1-ALPP	TRIM	Acetonitrile	4:20:01	PLE - HPLC(fluorescence detector)	(78)
zearalenone	Rye	CDHB	Bulk	1-ALPP	TRIM	Acetonitrile	4:20:01	PLE - HPLC(fluorescence detector)	(78)
zearalenone	Rice	CDHB	Bulk	1-ALPP	TRIM	Acetonitrile	4:20:01	PLE - HPLC(fluorescence detector)	(78)
fumonisin B1	Rice	Fumonisin B1	Bulk	DEAEM	TRIM	Acetonitrile	8:25:01	MISPE - LC-MS/MS	(79)
fumonisin B2	Rice	Fumonisin B1	Bulk	DEAEM	TRIM	Acetonitrile	8:25:01	MISPE - LC-MS/MS	(79)
fumonisin B3	Rice	Fumonisin B1	Bulk	DEAEM	TRIM	Acetonitrile	8:25:01	MISPE - LC-MS/MS	(79)
fumonisin B1	corn flake	Fumonisin B1	Bulk	DEAEM	TRIM	Acetonitrile	8:25:01	MISPE - LC-MS/MS	(79)
fumonisin B2	corn flake	Fumonisin B1	Bulk	DEAEM	TRIM	Acetonitrile	8:25:01	MISPE - LC-MS/MS	(79)
fumonisin B3	corn flake	Fumonisin B1	Bulk	DEAEM	TRIM	Acetonitrile	8:25:01	MISPE - LC-MS/MS	(79)
Ochratoxin A	Wheat	Molecularly imprinted polymer crushed monolith and bead and non-imprinted polymer (NIP) were provided by POLYINTELL research institute (Val de Reuil, France)*.						MISPE - HPLC	(80)
T-2 toxin	Maize	T-2 toxin	Bulk	mAAM	EGDMA	chloroform	12/60/1	MISPE - LC-MS/MS	(81)
T-2 toxin	Barley	T-2 toxin	Bulk	mAAM	EGDMA	chloroform	12/60/1	MISPE - LC-MS/MS	(81)
T-2 toxin	Oat	T-2 toxin	Bulk	mAAM	EGDMA	chloroform	12/60/1	MISPE - LC-MS/MS	(81)
zearalenone	Corn, Wheat	CDHB	Bulk Polymerization	(quinuclidin-3-yl methacrylate	TRIM	anhydrous acetonitrile	1:04:20	HPLC-UV/Vis	(83)
Six ergo alkaloids epimers	Barley	Metergoline	Suspension and Bulk Polymerization	MMA	ethylene glycol dimethyl acrylate	chloroform	1:06:24	LC-MS	(84)
five aflatoxins (M1,B1, B2, G1, G2 )	cereal-based baby food	5,7-Dimethoxycoumarin	Magnetic Core-Shell	MMA	EGDMA	Toluene/Methanol (90:10)	1:04:20	LC–MS/MS.	(85)
ochratoxin A	Rice	ochratoxin A	Core-shell	Dopamine	-	Tris-HCl buffer		Magnetic phase extraction	(86)
ochratoxin B	Rice	ochratoxin A	Core-shell	Dopamine	-	Tris-HCl buffer		Magnetic phase extraction	(86)
ochratoxin C	Rice	ochratoxin A	Core-shell	Dopamine	-	Tris-HCl buffer		Magnetic phase extraction	(86)
Zearalenone	Corn	Coumarin-3-carboxylic acid	Core-shell	MMA	EGDMA hydroxyethyl methacrylate	Ethanol		Solid phase extraction	(87)
Zearalenone	rice	Coumarin-3-carboxylic acid	Core-shell	MMA	EGDMA hydroxyethyl methacrylate	Ethanol		Solid phase extraction	(87)
Zearalenone	Wheat	Coumarin-3-carboxylic acid	Core-shell	MMA	EGDMA hydroxyethyl methacrylate	Ethanol		Solid phase extraction	(87)
Citrinin	Wheat	1-hydoxy-2-naphtoic acid	bulk polymerization	Acrylamide	Ethylene dimethacrylate	Acetonitrile		Solid phase extraction	(88)
Citrinin	Barley	1-hydoxy-2-naphtoic acid	bulk polymerization	Acrylamide	Ethylene dimethacrylate	Acetonitrile		Solid phase extraction	(88)
Fumonisin B1	maize	Fumonisin B1	Core-shell	See below^2^	-	H2O + Ammonium persulfate + tetramethylethylenediamine		Solid phase extraction	(89)
Aflatoxin G1	Corn	7-acetoxy-4-methylcoumarin	Core-shell	MMA	EGDMA	Ethanol		Solid phase extraction	(90)
Aflatoxin G2	Corn	7-acetoxy-4-methylcoumarin	Core-shell	MMA	EGDMA	Ethanol		Solid phase extraction	(90)
Aflatoxin B1	Corn	7-acetoxy-4-methylcoumarin	Core-shell	MMA	EGDMA	Ethanol		Solid phase extraction	(90)
Aflatoxin B2	Wheat	7-acetoxy-4-methylcoumarin	Core-shell	MMA	EGDMA	Ethanol		Solid phase extraction	(90)

Abbreviations: OTA: Ochratoxin A, MMA: methacrylic acid, EGDMA: Ethylene glycol dimethacrylate, 1-ALPP: 1-allylpiperazine, TRIM: trimethyltrimethacrylate, CDHB: cyclododecanyl-2, 4-dihydroxybenzoate, DEAEM: 2-(diethylamino) ethyl methacrylate, mAAM: methacrylamide, MISPE: molecularly imprinted solid phase extraction, PLE: pressurized liquid extraction, M/CL/T: Monomer/Crosslinker/Template ratio.

**Table 2:** Table of analytical method validation information

**Analyte**	**matrix**	**Template**	**MIP synthesis**	**extraction-analytical method**	**LOD (μg/L)**	**LOQ (μg/L)**	**Linear range (μg/L)**	**Recovery%**		**RSD%**	**Ref**	
α-zearalenol	Swine feed	CDHB	Bulk	PLE - HPLC(fluorescence detector)	0.7 to 1.3 ng/g	N.M	4–500 ng/g	88 to 97	2.1 to 2.4			(78)
α-zearalenol	Wheat	CDHB	Bulk	PLE - HPLC(fluorescence detector)	0.7 to 1.3 ng/g	N.M	4–500 ng/g	88 to 96	2.7 to 5.1			(78)
α-zearalenol	Corn	CDHB	Bulk	PLE - HPLC(fluorescence detector)	0.7 to 1.3 ng/g	N.M	4–500 ng/g	85 to 94	3.8 to 5.9			(78)
α-zearalenol	Barley	CDHB	Bulk	PLE - HPLC(fluorescence detector)	0.7 to 1.3 ng/g	N.M	4–500 ng/g	89 to 94	3.3 to 6.0			(78)
α-zearalenol	Rye	CDHB	Bulk	PLE - HPLC(fluorescence detector)	0.7 to 1.3 ng/g	N.M	4–500 ng/g	87 to 93	4.5 to 6.7			(78)
α-zearalenol	Rice	CDHB	Bulk	PLE - HPLC(fluorescence detector)	0.7 to 1.3 ng/g	N.M	4–500 ng/g	85 to 92	2.6 to 5.2			(78)
zearalenone	Swine feed	CDHB	Bulk	PLE - HPLC(fluorescence detector)	1.7 to 2.4 ng/g	N.M	6–500 ng/g	90 to 96	2.3 to 3.8			(78)
zearalenone	Wheat	CDHB	Bulk	PLE - HPLC(fluorescence detector)	1.7 to 2.4 ng/g	N.M	6–500 ng/g	89 to 97	3.8 to 4.1			(78)
zearalenone	Corn	CDHB	Bulk	PLE - HPLC(fluorescence detector)	1.7 to 2.4 ng/g	N.M	6–500 ng/g	86 to 92	3.1 to 5.4			(78)
zearalenone	Barley	CDHB	Bulk	PLE - HPLC(fluorescence detector)	1.7 to 2.4 ng/g	N.M	6–500 ng/g	90 to 96	3.9 to 5.2			(78)
zearalenone	Rye	CDHB	Bulk	PLE - HPLC(fluorescence detector)	1.7 to 2.4 ng/g	N.M	6–500 ng/g	86 to 94	4.1 to 5.6			(78)
zearalenone	Rice	CDHB	Bulk	PLE - HPLC(fluorescence detector)	1.7 to 2.4 ng/g	N.M	6–500 ng/g	87 to 92	3.1 to 4.6			(78)
fumonisin B1	Rice	Fumonisin B1	Bulk	MISPE - LC-MS/MS	N.M	N.M	N.M	66 to 89	1 to 9			(79)
fumonisin B2	Rice	Fumonisin B1	Bulk	MISPE - LC-MS/MS	N.M	N.M	N.M	62 to 75	1 to 8			(79)
fumonisin B3	Rice	Fumonisin B1	Bulk	MISPE - LC-MS/MS	N.M	N.M	N.M	67	2			(79)
fumonisin B1	corn flake	Fumonisin B1	Bulk	MISPE - LC-MS/MS	22 μg/kg	44 μg/kg	N.M	62 to 72	2 to 9			(79)
fumonisin B2	corn flake	Fumonisin B1	Bulk	MISPE - LC-MS/MS	4.5 μg/kg	9 μg/kg	N.M	71 to 75	2 to 8			(79)
fumonisin B3	corn flake	Fumonisin B1	Bulk	MISPE - LC-MS/MS	6 μg/kg	12 μg/kg	N.M	65 to 70	3 to 7			(79)
Ochratoxin A	Wheat	N.M	Micro-beads	MISPE - HPLC	N.M	N.M	N.M	82.5 to 102.7	1.5 to 4.5			(80)
T-2 toxin	Maize	T-2 toxin	Bulk	MISPE - LC-MS/MS	0.4 μg/kg	1.4 μg/kg	N.M	60 to 68	6 to 7			(81)
T-2 toxin	Barley	T-2 toxin	Bulk	MISPE - LC-MS/MS	0.5 μg/kg	1.7 μg/kg	N.M	63 to 73	3 to 4			(81)
T-2 toxin	Oat	T-2 toxin	Bulk	MISPE - LC-MS/MS	0.6 μg/kg	1.9 μg/kg	N.M	71 to 73	3 to 4			(81)
zearalenone	Wheat	CDHB	Bulk	MISPE-HPLC/UV-Vis	N.M.	3 μg/L	3- 9900 μg/L	Spike 20 µg/kg : 87%	0.062			(83)
zearalenone	Wheat	CDHB	Bulk	MISPE-HPLC/UV-Vis	N.M.	4 μg/L	3- 9900 μg/L	Spike 40 µg/kg : 82%	0.031			(83)
zearalenone	Wheat	CDHB	Bulk	MISPE-HPLC/UV-Vis	N.M.	5 μg/L	3- 9900 μg/L	Spike 85 µg/kg : 87%	0.025			(83)
zearalenone	Corn	CDHB	Bulk	MISPE-HPLC/UV-Vis	N.M.		3- 9900 μg/L	Spike 40 µg/kg : 86%	0.026			(83)
zearalenone	Corn	CDHB	Bulk	MISPE-HPLC/UV-Vis	N.M.		3- 9900 μg/L	Spike 85 µg/kg : 88%	0.018			(83)
zearalenone	Corn	CDHB	Bulk	MISPE-HPLC/UV-Vis	N.M.		3- 9900 μg/L	Spike 170 µg/kg : 90%	0.014			(83)
Six ergo alkaloids epimers	Barley	Metergoline	Suspension and Bulk	MISPE & MISPME- LC/MS	< 1µg/ kg	Em , Emn : 10 μg/kg	N.M	Em: Spiked After extraction : 79% , Spiked before extraction : 56%	Spiked After extraction: 9%, Spiked before extraction: 2%			(84)
Six ergo alkaloids epimers	Barley	Metergoline	Suspension and Bulk	MISPE & MISPME- LC/MS	< 1µg/ kg	Es:2 μg/kg		Es: Spiked After extraction: 71%, Spiked before extraction: 66%	Spiked After extraction: 7%, Spiked before extraction: 7%			(84)
Six ergo alkaloids epimers	Barley	Metergoline	Suspension and Bulk	MISPE & MISPME- LC/MS	< 1µg/ kg	Ecrn : 3 μg/kg		Et: Spiked After extraction: 71%, Spiked before extraction: 71%	Spiked After extraction: 6%, Spiked before extraction: 9%			(84)
Six ergo alkaloids epimers	Barley	Metergoline	Suspension and Bulk	MISPE & MISPME- LC/MS	< 1µg/ kg	The rest : 1μg/kg		Eco: Spiked After extraction: 65%, Spiked before extraction: 65%	Spiked After extraction 7%, Spiked before extraction: 3%			(84)
Six ergo alkaloids epimers	Barley	Metergoline	Suspension and Bulk	MISPE & MISPME- LC/MS	< 1µg/ kg			Ekr: Spiked After extraction: 68%, Spiked before extraction: 69%	Spiked After extraction: 14%, Spiked before extraction: 13%			(84)
Six ergo alkaloids epimers	Barley	Metergoline	Suspension and Bulk	MISPE & MISPME- LC/MS	< 1µg/ kg			Ecr: Spiked After extraction : 73% , Spiked before extraction : 68%	Spiked After extraction: 15%, Spiked before extraction: 7%			(84)
five aflatoxins (M1, B1, B2, G1, G2 )	cereal-based baby food	5,7-Dimethoxycoumarin	Magnetic Core-Shell	Magnetic based- LC/MS-MS	M1: 0.3 ng/kg	M1: 1 ng/kg	M1: 0.5-2 ng/kg	M1: 60%	> 10%			(85)
five aflatoxins (M1, B1, B2, G1, G2 )	cereal-based baby food	5,7-Dimethoxycoumarin	Magnetic Core-Shell	Magnetic based- LC/MS-MS	B1: 0.9 ng/kg	B1: 3 ng/kg	B1, B2, G1, G2: 2 -8 ng/kg	B1: 43%	> 10%			(85)
five aflatoxins (M1, B1, B2, G1, G2 )	cereal-based baby food	5,7-Dimethoxycoumarin	Magnetic Core-Shell	Magnetic based- LC/MS-MS	B2 :0.7 ng/kg	B2: 2.3 ng/kg		B2: 40%	> 10%			(85)
five aflatoxins (M1, B1, B2, G1, G2 )	cereal-based baby food	5,7-Dimethoxycoumarin	Magnetic Core-Shell	Magnetic based-LC/MS-MS	G1: 1 ng/kg	G1: 3.5 ng/kg		G1: 44%	>10%			(85)
five aflatoxins (M1, B1, B2, G1, G2 )	cereal-based baby food	5,7-Dimethoxycoumarin	Magnetic Core-Shell	Magnetic based- LC/MS-MS	G2 :1.7 ng/kg	G2 :5.8 ng/kg		G2: 39%	>10%			(85)
ochratoxin A	Rice	ochratoxin A	Core-shell	Magnetic phase extraction	1.8 pg·mL−1	-	0.01–1.0 ng·mL−1	0.71	0.023			(86)
ochratoxin B	Rice	ochratoxin A	Core-shell	Magnetic phase extraction	18 pg·mL−1	-	0.02–2.0 ng·mL−1	0.71	0.023			(86)
ochratoxin C	Rice	ochratoxin A	Core-shell	Magnetic phase extraction	3.2 pg·mL−1	-	0.002–0.2 ng·mL−1	0.71	0.023			(86)
Zearalenone	rice	Coumarin-3-carboxylic acid	Core-shell	Solid phase extraction	3.34 μg kg−1	10.00 μg kg−1	10.00-250 μg kg-1	0.901	0.0484			(87)
Zearalenone	Corn	Coumarin-3-carboxylic acid	Core-shell	Solid phase extraction	4.16 μg kg-1	12.5 μg kg-1	12.5-250 μg kg-1	0.848	0.0459			(87)
Zearalenone	Wheat	Coumarin-3-carboxylic acid	Core-shell	Solid phase extraction	2.09 μg kg-1	6.25 μg kg-1	6.25-250 μg kg-1	0.829	0.0462			(87)
Citrinin	Cereal	1-hydoxy-2-naphtoic acid	bulk	Solid phase extraction	1.5 μg L−1	5 μg L−1	5–4000 μg L−1	0.82	0.015			(88)
Fumonisin B1	maize	Fumonisin B1	bulk	Solid phase extraction	0.001 mg/kg	-	0.26–1.29 mg/kg	108–113%	0.55			(89)
Aflatoxin G1	Corn	7-acetoxy-4-methylcoumarin	Core-shell	Solid phase extraction	0.06 μg kg−1	0.2 μg kg−1	0.1-50 μg kg−1	82.6–116.7%	2.7-4.21%			(90)
Aflatoxin G2	Corn	7-acetoxy-4-methylcoumarin	Core-shell	Solid phase extraction	0.05 μg kg−1	0.15 μg kg−1	0.1-50 μg kg−1	82.6–116.7%	2.73-4.21%			(90)
Aflatoxin B1	Corn	7-acetoxy-4-methylcoumarin	Core-shell	Solid phase extraction	0.05 μg kg−1	0.2 μg kg−1	0.1-50 μg kg−1	82.6–116.7%	2.73-4.21%			(90)
Aflatoxin B2	Wheat	7-acetoxy-4-methylcoumarin	Core-shell	Solid phase extraction	0.06 μg kg−1	0.15 μg kg−1	0.1-50 μg kg−1	82.6–116.7%	2.73-4.21%			(90)

Abbreviations: N.M. not mentioned, OTA: Ochratoxin A, MMA: methacrylic acid, EGDMA: Ethylene glycol dimethacrylate,1-ALPP: 1-allylpiperazine, TRIM: trimethyltrimethacrylate, CDHB: cyclododecanyl-2,4-dihydroxybenzoate, MISPE: molecularly imprinted solid phase extraction, PLE: pressurized liquid extraction.

## Conclusion

This review has successfully highlighted the application of MIPs for the extraction, detection, and determination of mycotoxins in cereal samples. MIPs synthesized by different approaches have been introduced as a powerful tool in the sample extraction efficiency and selectivity enhancement over the target mycotoxins. The developed MIPs gained lower limits of quantification and detection (LOQ and LOD) and better extraction recoveries in comparison with conventional extraction and sample preparation methods (Table 2). 

MIPs offer high selectivity, reusability, stability, and low cost preparation.There is no doubt about practicality of the MIPs in extraction and sample preparation.However, template removal requires large amount of organic solvent and it is probably a time-consuming procedure. Future perspectives of MIPs can be improved synthesis with more efficient template removal and also capability of multi-analyte extraction, along with direct sample application in analysis method. 

## Author Contributions

The authors have equally contributed to the manuscript.
